# Haplotype diversity in the mitochondrial genome of the Egyptian river buffalo (*Bubalus bubalis*)

**DOI:** 10.1080/23802359.2020.1852622

**Published:** 2021-01-16

**Authors:** Nouran Adel Youssef, Manon Curaudeau, Soheir Mohamed El Nahas, Amal Ahmed Mohamed Hassan, Alexandre Hassanin

**Affiliations:** a Cell Biology Department, Genetic Engineering and Biotechnology Research Division, National Research Centre, Giza, Dokki, Egypt; b Institut de SYstématique, Évolution, Biodiversité (ISYEB), Sorbonne Université, MNHN, CNRS, EPHE, UA, Paris, France

**Keywords:** mtDNA genome, *Bubalus bubalis*, Egypt, Bangladesh, China, India, Italy, water buffalo

## Abstract

Here, we sequenced the complete mitochondrial genome of 29 Egyptian river buffaloes collected in two breeding stations of Egypt. The genome is 16,357–16,359 base pairs in length and contains the 37 genes found in a typical mammalian genome. The overall base composition is A: 33.1%, C: 26.6%, G: 13.9%, and T: 26.4%. Our analyses confirm that the mitochondrial genomes of swamp and river buffaloes are divergent (mean nucleotide distance = 2.3%), and show that Indian river buffalo haplotypes cluster into three haplogroups, named RB1, RB2, and RB3 (mean distance = 0.25–0.26%) and that the 24 Egyptian buffalo haplotypes fall into RB1 (with the Bangladeshi, Chinese and Italian buffalo haplotypes) and RB2.

The domestic water buffalo (*Bubalus bubalis*) is found on all continents inhabited by humans, with a total population of 202 million heads (Zhang et al. 2020). There are two morphological types: (a) the river buffalo, which is mainly used for milk production, is black with horns showing a double curvature (at first, they are directed downward and backward, and then curl upward in a spiral); (b) whereas the swamp buffalo, which is primarily used as a draft animal, is generally dark gray with white chevrons on the throat, white socks, and semi-circular horns that always remain approximately in the same plane as the forehead (MacGregor 1941; Zhang et al. 2020). The two types have been domesticated independently: the river buffalo in the western region of the Indian subcontinent *ca*. 6300 years BP, and the swamp buffalo in the China/Indochina border region *ca*. 3000–7000 years BP (Zhang 2020). The river buffalo was introduced to Egypt from India via Mesopotamia during the nineth century (Sidky 1951). Today, it is the most important domestic animal of Egypt, with approximately 3.7 million heads (FAO 2017) used mainly for milk, but also for meat and as a draft animal.

In this study, we sequenced the mitochondrial genome of 29 Egyptian river buffaloes and made a comparison with all sequences of *Bubalus* available in GenBank. As indicated in Figure 1, blood samples were collected in two breeding stations: Kafr El Sheikh for the buffaloes from northern governorates, and Beni Suef for the buffaloes from southern governorates. Total DNA was extracted following the phenol-chloroform protocol published in Sambrook and Russel (2001). DNA extracts are stored in the ISYEB research collection at the Muséum national d’Histoire naturelle (Paris, France). Three overlapping PCR products were amplified using the primers published in Hassanin et al. (2012, 2020): (1) GluMA and LMet3-CH; IleU and Leu2LM1-CH; and (3) Ser2U and LPro-CH. PCR reactions were performed as detailed in Hassanin et al. (2020). The amplicons were sequenced using the Ion Torrent Personal Genome Machine (Thermo Fisher Scientific). The NGS reads were assembled on Geneious® 10.2.2 (Biomatters Ltd.) using the mitochondrial genome reference for *Bubalus bubalis* (accession number: NC_006295). The 29 new mitochondrial genomes generated for this study were annotated on Geneious and deposited in GenBank.

**Figure 1.**
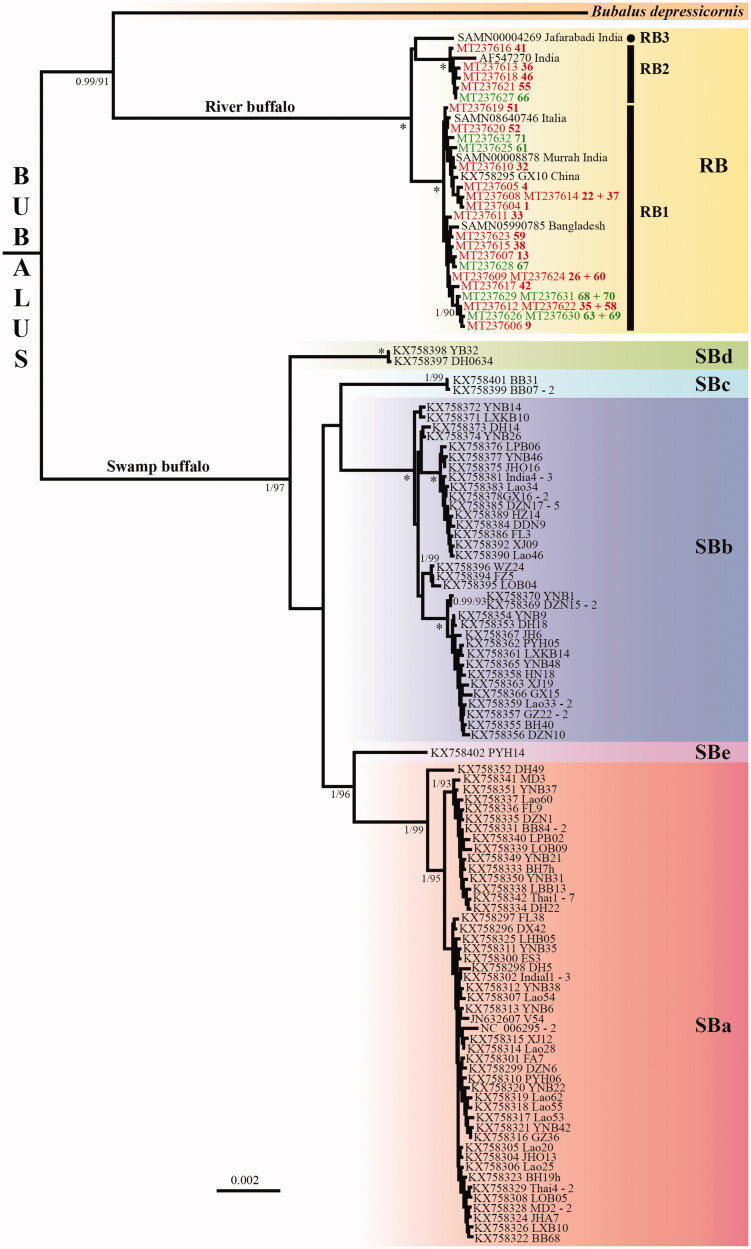
Phylogeny of *Bubalus* based on complete mitochondrial genomes. The tree was reconstructed under MrBayes using the 118 mitogenomic haplotypes of *Bubalus* identified in our alignment of 16,356 nucleotides. The tree was rooted with *Syncerus* (not shown). Haplogroups showing more than 0.5% of nucleotide divergence are highlighted by different colored rectangles, and those found in the swamp buffalo are named SBa to SBe (the name of the haplotype is followed by a dash and a number when the same haplotype was found in at least two individuals). Among the 29 mitogenomes of Egyptian river buffalo, the 21 samples collected in Kafr El Sheikh are indicated in red, whereas the eight samples collected in Beni Suef are indicated in green. The four mitogenomes of river Buffalo assembled using SRA data available in GenBank are named with the SAMN accessions. For nodes supported by bootstrap percentage (BP) ≥ 90 in the RAxML analysis (see details in Material and Methods), the two values correspond to the posterior probability (PP calculated under MrBayes, left of the slash) and BP (right of the slash). An asterisk is used when both MrBayes and RAxML analyses provided maximal support values, i.e. PP = 1 and BP = 100, respectively. No information was provided for nodes supported by BP < 90.

The mitochondrial genome of the Egyptian buffalo is a circular double-stranded DNA sequence that is 16,357–16,359 base pairs in length. The 29 mitogenomes of Egyptian buffalo represent 24 haplotypes. The overall base composition is A: 33.1%, C: 26.6%, G: 13.9%, and T: 26.4%. The genome contains the 37 genes found in a typical mammalian genome. All protein-coding genes of the mtDNA have a methionine start codon (ATR), except ND4L (GTG) and ND2 (ATT for members of haplogroup RB2, see below). With the exception of the CYTB gene, which has the stop codon AGA, all protein-coding genes appear to be terminated by TAA or TAG. However, this stop codon is incomplete in the COX3, ND3 and ND4 genes. The control region is 926-928 bp in length.

The 24 mitochondrial haplotypes detected for the Egyptian buffalo were compared to the mitogenomes available in GenBank for the genus *Bubalus*, representing two river buffaloes, 112 swamp buffaloes and the anoa (*Bubalus depressicornis*). We also assembled four mitogenomes of Indian river buffalo using SRA (Sequence Read Archive) data (GenBank accession numbers are detailed in Figure 1). *Syncerus caffer* (accession number: NC_020617) was used as an outgroup. After exclusion of identical haplotypes and removal of ambiguous regions for primary homology, our final DNA alignment is 16,356 bp in length and contains 119 sequences, including 30 haplotypes for the river buffalo and 87 haplotypes for the swamp buffalo.

The tree shown in Figure 1 was reconstructed with MrBayes 3.2.6 (Ronquist et al., 2012) and the GTR + I + G model. The Bayesian posterior probabilities (PP) were calculated using 10,000,000 Metropolis-coupled MCMC generations, tree sampling every 1000 generations, and a burn-in of 25%. Bootstrap percentages (BP) were calculated using RAxML version 8.2.10 (Stamatakis, 2014), with 25 rate categories (CAT approximation), 1000 bootstrap replicates, and the GTR model. The phylogenetic analyses support the paraphyly of the species *B. bubalis* since the anoa (*B. depressicornis*), a wild species endemic to Indonesia, is found to be the sister-group of the river buffalo (PP = 0.99; BP = 90), with the swamp buffalo at the outside. As previously pointed out in Hassanin et al. (2012), this result suggests that river buffalo and swamp buffalo belong to distinct species. Nuclear data are however needed to validate this taxonomic proposition. Another intriguing result concerns the levels of nucleotide divergence found in the two types of water buffalo. In agreement with the analyses of Wang et al. (2017), the 87 haplotypes of swamp buffalo cluster into five haplogroups (here named SBa, SBb, SBc, SBd and SBe) showing more than 0.5% of nucleotide divergence. By contrast, all the 30 haplotypes detected for the river buffalo belong to a single main haplogroup, here named RB. Using a threshold < 0.5%, the river buffalo haplotypes can be further divided into three subgroups (mean distance = 0.25–0.26%): RB1 includes 23 haplotypes found in Egypt, Bangladesh, China, India and Italy; RB2 contains six haplotypes found in Egypt and India; and RB3 is only represented by the sequence of the Indian Jafarabadi breed. The results suggest therefore that the Egyptian buffalo livestock did not derive from a unique breed imported from Mesopotamia or India, but rather from multiple migrants, a hypothesis previously mentioned by Hassan et al. (2009) based on D-loop sequences.

## Data Availability

The data that support the findings of this study are openly available at https://www.ncbi.nlm.nih.gov/genbank/ (accession numbers MT237604-MT237632) and https://osf.io/kt84f/ (DNA alignment used for phylogenetic analyses).
